# Better public decisions on COVID-19: A thought experiment in metrics

**DOI:** 10.1016/j.puhip.2021.100208

**Published:** 2021-10-29

**Authors:** David J. Tonjes, Krista L. Thyberg, Elizabeth Hewitt

**Affiliations:** Waste Data and Analysis Center, Department of Technology and Society, Stony Brook University, New York, USA

**Keywords:** Disease metrics, Decision support, Public involvement, Infectious population, Odds ratio

## Abstract

**Objectives:**

Poor decision-making is a hallmark of the COVID-19 pandemic. Better metrics would help improve decision-makers' understanding of the scope of the pandemic and allow for better public understanding/review of these decisions.

**Study design:**

Two novel metrics of disease impact were compared with more commonly used standard metrics.

**Methods:**

A multi-criteria decision analysis technique, used previously to support metric selection in solid waste planning, was adapted to compare number of deaths, hospitalisations, positive test results and positivity rates (standard COVID-19 impact metrics) with a simple model that estimates the total number of potentially infectious people in an area and an associated odds ratio for infectious people.

**Results:**

The odds ratio and total infectious population estimate metrics scored better in a comparison analysis than number of deaths, hospitalisations, positive test results and positivity rates (in that order).

**Conclusions:**

The novel metrics provide a more effective means of communication than other more common measures of the outbreak. These superior metrics should support decision-making processes and result in a more informed population.

## 1 Introduction

Decision-making during the COVID-19 pandemic has been suboptimal and metrics conveyed to the public have contributed to this greatly. Risk perception varies from risk computation [1] and how risks are presented impacts perception [2]. Previous work in another field of study shows that use of better metrics is beneficial for both decision makers and affected populations [3], which should also be true for the COVID-19 pandemic. Here, two alternative metrics are presented to supplement the standard set of COVID-19 incidence indicators. These novel metrics are a derived estimate of the total number of infectious people and an odds ratio for the area population, developed from the total number of infectious people.

## 2 Metric choices

Prior research compared solid waste measures using a multi-criteria decision analysis technique [3]. Although managing garbage and the pandemic are seemingly unrelated, both require interdisciplinary understanding and measurements to reach good decisions. A set of attributes can help inform which metrics are needed for good public decision-making, and these attributes were used to consider six different evaluation metrics for the pandemic (Table 1).

**Table 1:** COVID-19 metrics comparison (1–3 scale: 1 = poor, 2 = good, 3 = excellent).

Metric	Direct	Objective	Clear	Comparable	Practical	Reliable	Useful	Relevant	Error	Policy Relevant	Score
	Tracks result it intends to measure	No ambiguity in measurements; clearly defined (uses common definitions)	Simple, easy to interpret	Allows for areal-temporal comparisons	Data obtained timely with reasonable costs	Data sufficient, dependable, and consistent for decision-making	Meaningful measure of system change; useful for daily decision making	Provides high priority information; communicates system information	Errors do not propagate	Relevant to current health policies	
Positive tests	2	2	3	2	2	2	2	3	1	1	20
Positivity rate	1	1	1	3	2	1	1	2	1	2	15
Hospital-isations	2	3	3	2	1	2	2	3	3	3	24
Deaths	2	2	3	2	1	2	2	3	2	3	22
Total no. of infections	3	2	3	2	2	3	3	3	1	3	25
Infection odds ratio	3	2	3	3	2	3	3	3	1	3	26

### 2.1 Deaths

Reports on deaths are readily understood, but quantifying deaths can be inconsistent. Currently, reports of deaths from COVID-19 are controversial because confusion surrounds how to count deaths as a result of underlying causes in conjunction with COVID-19. Deaths can be a powerful measure, but are retrospective in nature because they lag other measures. For instance, in Suffolk County (NY, USA) (where the authors are located), the first death occurred on 16 March 2020, but by then awareness of community spread was widespread and the state lockdown was implemented within 5 days. For most areas of the country, deaths were few at the start of the pandemic, especially compared with other established health threats, which led to the perception that the threat of COVID-19 was minimal. However, by October 2020, over 200,000 deaths had been recorded nationwide. The death rate has also been decreasing over time, which may diminish its value as a measure of pandemic intensity.

### 2.2 Hospitalisations

Hospitalisations are a better measure of the pandemic with less lag time from the initial infection. Hospitalisation of an infected individual demonstrates an acute health problem and is readily understood as such by the public. However, peak values for hospitalisations vary (for instance, Suffolk County has 3400 hospital beds) and hospitalisations disguise unintended consequences, such as when COVID-19 patients displace patients with other conditions. Hospital bed counts are not intuitive (i.e. few people know how many beds exist until they are nearly full) and hospitalisations only apply to a subset of the infected population as the majority do not require hospitalisation. The percentage of infected people requiring hospitalisation has decreased with changing disease demographics.

### 2.3 New infections

The number of newly detected cases is readily understandable and the most widely reported metric. However, this measure is flawed, limited and partially defined by test availability. In the early stages of the pandemic, not everyone suspected of infection could be tested. Now, some reports of increasing infections are being driven by increases in testing. Many individuals infected by SARS-CoV-2 are not tested, so new case counts are underestimates of disease incidence. In addition, test results can be delayed because laboratories are working above capacity, so the daily reports actually reflect several days' results. This purported point-in-time datum does not measure any single day's impact on the pandemic.

### 2.4 Positivity rate

The ‘positivity’ rate is used to track infection intensity. It divides the number of positive cases by the total number of tests, intending to minimise the effect of increasing test availability. However, the denominator (number of tests) is affected by test availability, and the numerator (positive cases) primarily results from two sets, those fearing they are infected or those required to prove non-infection. One concentrates the rate, the other dilutes it. At best, the positivity rate asymptotically approaches true SARS-CoV-2 incidence rate as testing rates approximate a random sample of the population. The numerator draws from several days' results due to reporting lags, so the daily positivity rate applies to no specific day. And positivity rate is obscure. Why is 5% (or 8%) meaningful? Is a change from 1% to 2% actionable? Does a positivity rate of 10% describe a pandemic impact ten times that of 1%? Its direct meaning is murky.

### 2.5 Estimate of the number of infectious people

This metric is impossible to generate directly (unlike deaths, hospitalisations or positive test results) and is the output of a simple model. Positive test results reflect about 1 in 10 actual infections [4]. Thus, when Suffolk County reports 40 positive tests in a day, this suggests that there are about 400 newly infected individuals. Since people are infectious for about 10 days [5], the sum of infectious people is the number of newly infected people on the target day, plus the cumulative total of new infections over the previous 9 days. For Suffolk County, where ∼40 new infections have been reported since June, multiply the total of daily infected people by 10 – implying there are about 4000 infected people capable of spreading the disease at any particular time. This is about 0.3% of the County's 1.45 million population. Restricting reported infections to adults (since children are not tested as often) means about 3.3% of adults are infectious. Another example is in Florida between 5 and 14 July 2020, where the sum of new infections was 102,372 (with widely varying numbers each day), implying, as of 14 July 2020, approximately 1 million people were potentially infectious (because the reported infections are only one-tenth of all infections); thus, 6% of the adult population in Florida was potentially infectious on 15 July 2020.

This modelling measure is an integral of 10 days of results, which blurs the effect of reporting delays and lags of results, although it is actually a point-in-time measure. Reporting the overall number of infectious people more accurately describes the public health task. For example, in Suffolk County, the true task in COVID-19 suppression is not contact tracing the 40 new cases from testing each day, but instead finding and isolating the pool of 4000 people capable of spreading COVID-19.

### 2.6 Infectious population ratio

The infectious population ratio relates directly to the modelled estimate of the number of infectious people. For Suffolk County, 40 new detected infections per day translates to 4000 potentially infectious people, which is 1 in 300 adults. For the state of Florida on 15 July 2020, 1 in 17 adults were potentially infectious. These data are impactful: most people will visualise a pool of 17 or 300 people and can determine if this is risky. For decision-makers, the scope of infections becomes clearer and they can apply these values to various scenarios to determine how many people may be potentially infectious.

## 3 Discussion

The analysis in Table 1 underscores that the current reliance on communicating positive test results to the public is not helpful in reaching/making better decisions and, more importantly, is not supportive of public perceptions and review of these decisions. Conversely, the ranking scores, as a result of the metric comparison analysis (see Table 1), supports the use of the odds ratio value for better risk determinations. For instance, if 1 in 300 adults is potentially infectious and, assuming the average school employs 100 adults, decisions-makers can then discuss relative risks of opening schools in clear and obvious ways, and the people affected by the decisions (i.e. teachers, staff and parents) can in turn envision their individual risks. This is true for similar reopening debates, where knowing the infectious odds ratio allows individuals to weigh the chances of encountering an infectious person.

Describing the peak of the pandemic in Florida as a time when 1 in 17 adults was infectious is as visceral as reports of overfull emergency rooms and intensive care units and 100 or more daily deaths. For some, a COVID-19 death rate of 100 per day seems small in the perspective of a population of more than 21 million. However, when the odds of encountering an infectious person are presented as 1 in 17, this is a compelling depiction of the risky nature of social interactions. In addition, the odds ratio metric gives meaning to public health initiatives such as calls for universal mask use, especially where infection rates are high.

The parameter model can be modified to account for evolving data about COVID-19. Perhaps the ratio of tested to untested infectious people is lower than 1 in 10 [6] or even as low as 2 in 5 [7] (given a 60:40 ratio of symptomatic to asymptomatic infections [8]). Perhaps the length of the infectious period is less than 10 days [9]. Perhaps not all positive tests mean infectious potential [10], so a divisor term should be used to transform the new infections count to the number who are infectious. The values are not as important as the ability for the metrics to be readily understood and used by officials and the general public.

## 4 Conclusions

Individuals' risk perception differs from risk assessors' mathematical models [1] and these interpretations are not always logical or mathematically coherent [2]. The number of SARS-CoV-2 infectious people in a region and its associated odds ratio are readily comprehensible data. Having such evaluations of the COVID-19 pandemic will help guide officials to make more relatable and transparent decisions. These metrics also clearly outline the overall scope of infections in a community, enabling public health goals to be better tuned to current conditions. Describing the pandemic's local status this way can bring clarity to the general public about action plans made by decision-makers.

## Ethical approval

No institutional ethical approvals were sought or needed for this study because there were no human or animal subjects in the experiment.

## Funding

This research did not receive any specific grant from funding agencies in the public, commercial, or not-for-profit sectors.

## Competing interests

The authors report no conflicts of interest.
